# “QuanTour” illuminates Europe with single photons: celebrating the International Year of Quantum Science and Technology 2025

**DOI:** 10.1038/s41377-025-01745-w

**Published:** 2025-02-17

**Authors:** Fei Ding

**Affiliations:** Institut für Festkörperphysik, Leibniz Universität Hannover, Appelstraße 2, 30167 Hannover, Germany

**Keywords:** Single photons and quantum effects, Quantum optics

## Abstract

Witness the journey of a solid-state quantum light source travelling across Europe and beyond—like an Olympic torch relay in the quantum realm.

With the United Nations’ proclamation of 2025 as the “International Year of Quantum Science and Technology” (IYQ) (https://quantum2025.org/en/), public attention is turning to the transformative potential of quantum technologies. At the heart of these advancements lies the controlled generation of quantum light states—key to quantum communication, computing, sensing, and metrology.

One remarkable initiative leading up to the IYQ is **QuanTour**, an ambitious project that has been shining a spotlight on semiconductor photonic nanostructures and single-photon generation since April 2024 (https://www.quantum2025.de/quantour). At its core is cutting-edge science: a single semiconductor quantum dot embedded in a micro-sized, dartboard-shaped nanophotonic cavity [Figs. 1 and 2]. The intricate fabrication process and exceptional quantum optical properties of this source were recently detailed in publications by the organizers (10.1021/acsphotonics.4c01873), (10.1515/nanoph-2024-0519). Known as circular Bragg gratings, these microcavities feature high photon-extraction efficiency across a broad spectral range and strong light confinement. At the center of this “micro-dartboard,” a quantum emitter generates single photons on demand—a vital resource for building the future quantum internet (10.1063/1.3615051). This innovation is not only highly significant for fundamental research on single-photon sources but has also recently demonstrated its potential for real-world intercity quantum key distribution (10.1038/s41377-024-01593-0, 10.1038/s41377-024-01488-0).

**Fig. 1:**
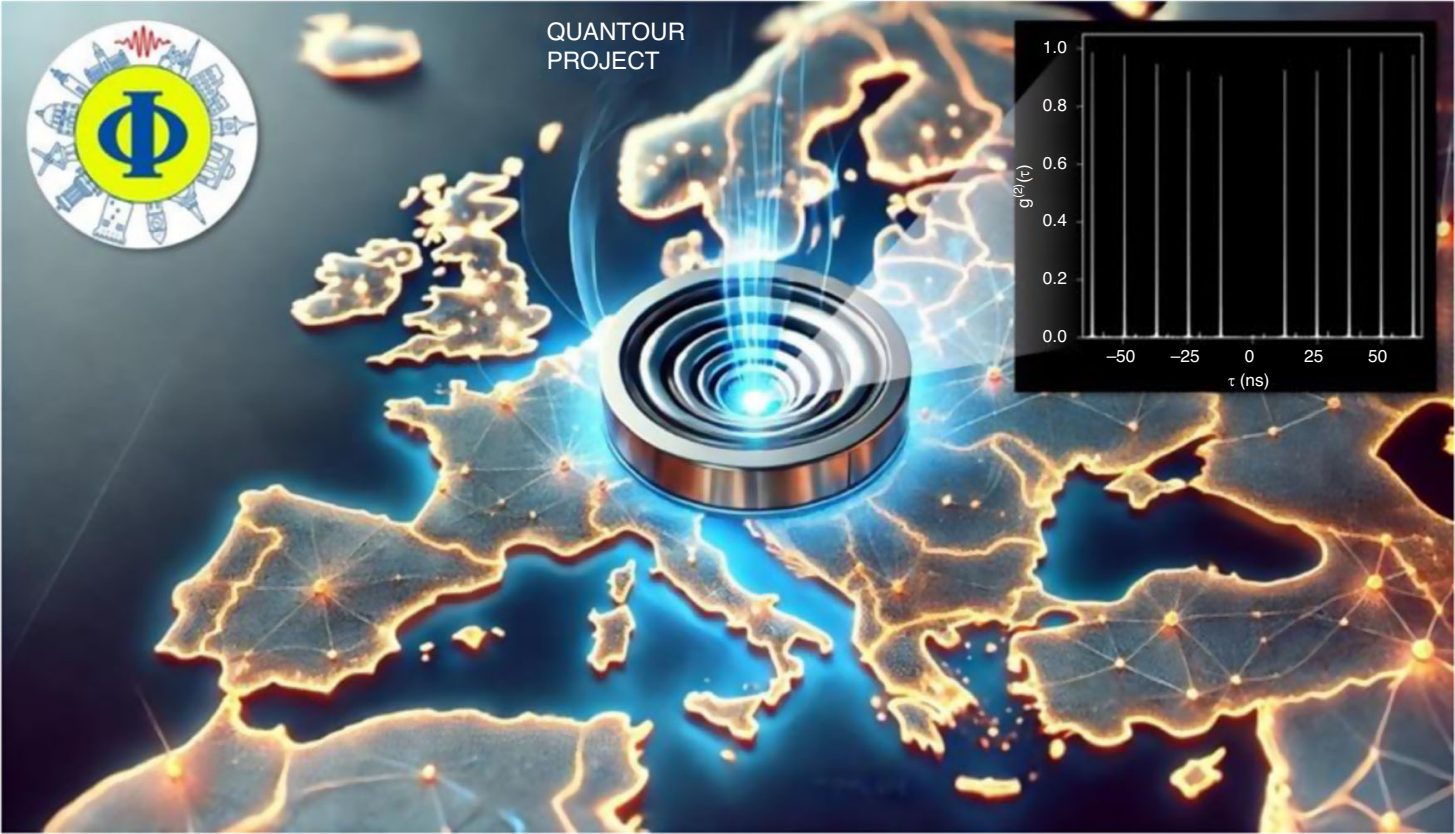
In a display reminiscent of an Olympic torch relay, the semiconductor-based, portable single photon source travels across Europe and beyond. The inset shows the autocorrelation function measured with this device, indicating the near-perfect single photon emission characteristics

**Fig. 2:**
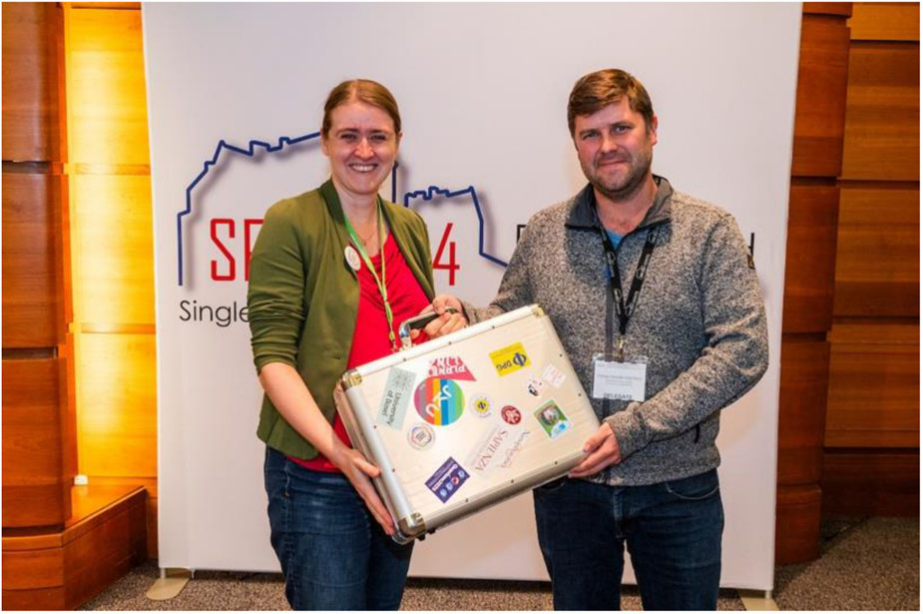
The QuanTour project is spearheaded by Doris Reiter (TU Dortmund) and Tobias Heindel (TU Berlin) in collaboration with the German Physical Society (DPG)

The QuanTour project is spearheaded by Doris Reiter (TU Dortmund) and Tobias Heindel (TU Berlin) in collaboration with the German Physical Society (DPG) [Fig. 2]. Tobias specializes in quantum communication and solid-state devices (10.1002/qute.202100116), while Doris is renowned for her theoretical contributions to solid-state quantum optics and the SUPER scheme (10.1103/PRXQuantum.2.040354). Together, they are working with over 14 leading researchers worldwide to bring QuanTour to life.

In a display reminiscent of an Olympic torch relay [Fig. 3], the quantum light source travels across Europe and beyond, housed in a sleek, code-secured suitcase. At each stop, host research teams perform correlation measurements to validate the single-photon generation. So far, the quantum source has visited eight laboratories, demonstrating Europe’s scientific community’s dedication to pushing the boundaries of quantum research.

**Fig. 3:**
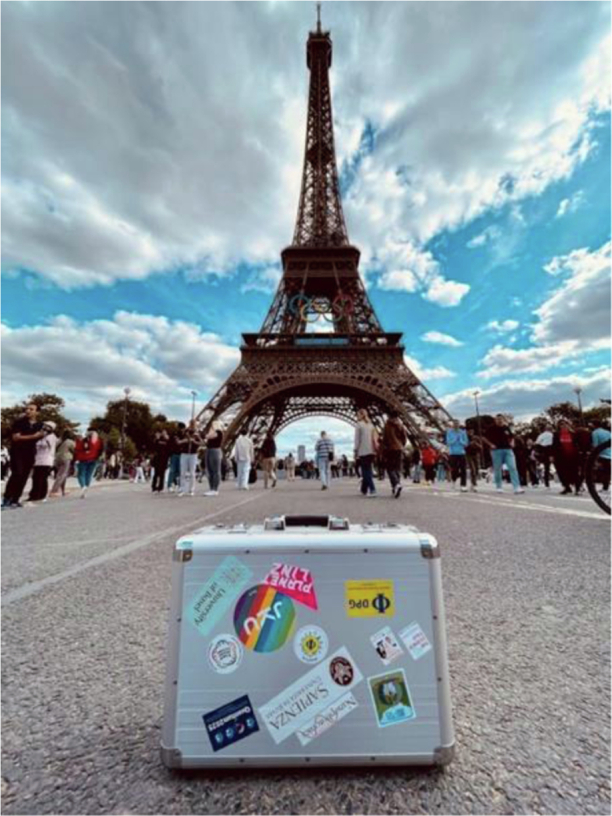
The single-photon source, securely housed in a sleek, code-locked suitcase, traveled to Paris and was sitting in front of the iconic Eiffel Tower adorned with Olympic rings

But QuanTour doesn’t just stay in the lab—it aims to captivate the public, especially younger audiences, by showcasing the excitement of quantum technology. Through Instagram videos (@quantour.eu) (https://www.instagram.com/quantour.eu/), the project offers a glimpse into leading quantum research labs, the underlying technology, host cities, and research teams. With humor and creativity, the content includes highlights like “Quantum Security” and a “Sticker Ceremony,” where each destination adds its emblem to the traveling suitcase.

To enhance outreach, QuanTour has teamed up with **TheScienceTalk** to produce podcasts featuring interviews with hosting researchers (https://thesciencetalk.com/quantour/), diving deeper into the physics and people behind the project. This blend of engaging and informative content fosters broader interest in semiconductor physics and quantum science.

As QuanTour continues its journey, it serves as both a platform for scientific collaboration and a bridge to public engagement. By showcasing quantum light sources and fostering international cooperation, the project lays the groundwork for the IYQ, inspiring future innovations in quantum science and technology.

